# Organoleptic and Physicochemical Properties of Soy‐Milk Yoghurt Enriched with *Moringa Oleifera* Root Powder

**DOI:** 10.1002/gch2.202100097

**Published:** 2022-02-23

**Authors:** Roger Ponka, Peter Mukong Zhung, Gaston Zomegni, Carolin Gabriel Tchouape, Elie Fokou

**Affiliations:** ^1^ Department of Agriculture Livestock and Derivated Products National Advanced School of Engineering of Maroua University of Maroua Maroua PO BOX 46 Cameroon; ^2^ Department of Textile and Leather Engineering National Advanced School of Engineering of Maroua University of Maroua Maroua PO BOX 46 Cameroon; ^3^ SNV‐Presec Maroua Maroua PO BOX 46 Cameroon; ^4^ Department of Biochemistry Faculty of Science University of Yaoundé I Yaoundé PO BOX 812 Cameroon

**Keywords:** cow milk, *moringa* root powder, physicochemical properties, soy yoghurt

## Abstract

The organoleptic and physicochemical properties of soy‐milk yoghurt enriched with moringa root powder are evaluated here. *Moringa oleifera* soy‐milk yoghurt is produced at different formulations blended from cow milk: soymilk: *Moringa* in a ratio of A (100%:0%:0%; B (60%:39.9%:0.1%); C (50%:49.9%:0.1%), D (40%:59.9%:0.1%), and E (0%:99.9%:0.1%) with sample A serving as the control. Sensory analysis is done for each formulation and the physicochemical properties of the preferred formulations are performed. The pH and titratable acidity are measured by a pH meter and titration respectively. Proximate composition is measured according to Association of Official Analytical Chemists methods. The mineral content is determined by atomic spectrophotometry. Results show that soy‐milk *Moringa* enriched yoghurts (B and C) are preferred after choosing the control (A) as the best. The incorporation of the soy‐milk *Moringa* significantly increases the fat, fiber, protein, copper manganese, and iron content in the samples (*p* < 0.05). Thus Moringa enriched soy‐milk yoghurts (B and C) represent a cheaper alternative to the control (A) providing protein‐energy for low income families that is expected to help reduce the occurrence of kwashiorkor and wasting. The presence of zinc and calcium is expected to help in bone growth and development hence preventing stunting in children under five years and iron is expected to help reduce the prevalence of anemia among pregnant women.

## Graphical abstract

The incorporation of *Moringa Oleifera* root powder into soy‐milk joghurt significantly increases the fat, fiber, protein, copper manganese, and iron content in the samples. Thus Moringa enriched soy‐milk yoghurts (B and C) represent a cheaper alternative to the control (A) providing protein‐energy for low income families that is expected to help reduce the occurrence of kwashiorkor and wasting.

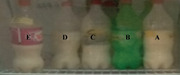

## 1 Introduction

Today, the estimated number of undernourished people worldwide has increased to 815 million^[^ ^1^ ^]^ despite the numerous interventions for the amelioration of socioeconomic status and food security in Africa. In Africa in general and West Africa in particular, the prevalence of undernourishment is 20.0% and 11.7%, respectively.^[^ ^1^ ^]^ In Cameroon, the nutritional status keeps deteriorating.^[^ ^2^ ^]^

The prominent cause of stunting in Cameroon is zinc deficiency as reported by Anyangwe^[^ ^3^ ^]^ and is known to affect 33% of children under the age of 5 years. Also anemia (caused by iron deficiency) was reported to be high (49.3%) among pregnant women, by the World Health Organisation.^[^ ^4^ ^]^ Protein deficiency is aggravated by bland, starchy staple foods especially in the northern regions with corn and millet serving as the main staple foods.^[^ ^2^ ^]^ These protein deficient meals present the risk of protein malnutrition (kwashiorkor) in children that need to be well fed in the first 1000 d of their lives.^[^ ^5^ ^]^ This suggests the need for food programs that intervene in curbing malnutrition rates.^[^ ^6^ ^]^

Yoghurt can serve as a good source of protein in order to prevent kwashiorkor especially to weaned children below 5 years old. Commercial yoghurt is relatively expensive when compared to soy yoghurt due to soymilk substitution of cow milk. Composite soy‐milk yogurt is thus a relatively inexpensive source of protein‐energy as well as micronutrients upon soymilk substitution.^[^ ^7^ ^]^

Recent increase in snacking within low and middle income countries such as Asia, Latin America and Africa,^[^ ^8^ ^]^ suggests that a possible intervention could be to fortify snacks (such as yoghurt). The fortification potential of *Moringa oleifera* has been studied more in the leaves than its roots. Kurana and Rajni^[^ ^9^ ^]^ show that the roots are also of nutritional importance. *Moringa oleifera* root powder (MRP) possessing minerals and macronutrients can serve as a novel route alongside soymilk to increase the nutrient content of widely consumed foods/snacks such as yoghurt. However care must be taken to ensure the complete removal of the bark of moringa roots that has substances such as alkaloids that are known to be poisonous if consumed in excess.^[^ ^10^ ^]^

In Sub‐Saharan countries, the *Moringa oleifera* tree is under‐utilized as a food source whilst it is considered a “miracle tree” because of its nutritive value.^[^ ^11^ ^]^ Improving its agriculture‐nutrition nexus as intended by this study will create novel nutritional interventions, able to ensure the fulfillment of the adequacy of micronutrients as well as protein‐energy, needed especially for poor and rural areas in combating malnutrition and increasing food security.

Food fortification is one of the key strategies for a durable fight against different types of malnutrition including protein and micronutrient deficiencies. It involves the introduction of the food supplement through an industrial product or locally processed food that is largely consumed such as traditional yoghurt (also known locally in most parts of Cameroon as *Kossam*. Giving that composite soy milk yoghurt is an alternative of cow milk yogurt that is relatively inexpensive and even richer in protein due to the substitution of cow milk with soy milk.^[^ ^12^ ^]^ It will serve as a good route in valorizing moringa root that is essentially rich in micronutrients such as zinc, iron, and magnesium.^[^ ^9^ ^]^

Soymilk substitution provides cheap protein to prevent the risk of kwashiorkor and import micronutrients needed for proper growth and development of children below five years. This in turn will galvanize the fight against malnutrition such as stunting especially in weaned children below five years and anemia in women of child bearing age.^[^ ^9^ ^]^ Also soymilk substitution could help reduce the selling price of yoghurt and hence increase the spectrum of purchase even to low income and poverty stricken families. Soymilk essentially rich in iron will help increase iron beneficial against anemia due to lack of iron.

Prevalent malnutrition in Cameroon caused by undernourishment, presents a dire need for food supplementation programs that will help increase the micronutrient content of food (food fortification). In this case moringa soy milk enrichment of yoghurt has been studied as a possible route for mineral fortification and concomitant cheaper source of protein to those who cannot afford cow milk yoghurt due to high prices.^[^ ^7^ ^]^

The aim of this study was to evaluate the organoleptic and physicochemical properties of soy‐milk yoghurt enriched with moringa root powder.

## 2 Experimental Section

### 2.1 Raw Materials

The root of *Moringa oleifera*, soybean, fresh row cow milk, and other ingredients were collected, in Maroua, a town in the Far North region of Cameroon. This town is located between latitude 10° and 13° North and between longitude 13° and 16° East. Its population is estimated at about 200 000 inhabitants.^[^ ^13^ ^]^ All ingredients were transported in the food technology laboratory at Institut de Recherche Agronomique pour le Développement (IRAD)‐Maroua.

### 2.2 Preparation of Raw Materials

#### 2.2.1 Production of Moringa Root Powder

Mature *Moringa* root was harvested from the parent plant and cut into smaller pieces. It was then washed in water and its skin stripped off and the peeled roots cut into smaller pieces. The shredded pieces were then dried under sunlight (40–44 °C) for 3–4 d.^[^ ^14^ ^]^ Dried pieces of *Moringa* root were milled into powder, sieved, and stored in polyethylene bags.

#### 2.2.2 Production of Soymilk Equivalent

Soy milk was prepared according to procedures gleaned from related studies such as that of Osudahunsi et al.^[^ ^15^ ^]^ and that of Akusu and Wordu^[^ ^12^ ^]^ and simplified as follows:

Cleaned soybean seeds (0.5 kg of yellow variety) were soaked in 2 L of water (25 °C; 24 h). Blanching was done by boiling for 5 min after which they were drained. The blanched soybeans were ground in a blender. The slurry was filtered at ratio 1:6 of water to slurry through muslin cloth to obtain filtrate. Filtrate was simmered for 20 min to obtain soymilk.

#### 2.2.3 Formulation of Composite Moringa‐Soy Milk Enriched Cow Milk

Here sample A was formulated to serve as the positive control (standard) used to choose the best alternative from samples B, C, D, and E, that is preferred following sensory analysis.

Fresh cow milk was then filtered using muslin cloth in the food technology laboratory at IRAD‐Maroua. The filtered milk was used alongside soy milk and *Moringa* root powder in formulations as shown in **Table** **1**.

**Table 1:** Different formulations of composite soy milk and Moringa

Samples^a)^	Whole cow milk [%]	Soy milk [%]	*Moringa* root powder [%]	Sugar [g/100 mL]
A (Control)	100	0	0	7.5
B	60	39.9	0.1	7.5
C	50	49.9	0.1	7.5
D	40	59.9	0.1	7.5
E	0	99.9	0.1	7.5

^a)^ A = 100% cow whole milk + 7.5 g sugar/100 mL ( control); B = 60% whole cow milk + 39.9% soy milk + 0.1% *Moringa* root powder; C = 50% whole cow milk + 49.9% soymilk + 0.1% *Moringa* root powder; D = 40% whole cow milk + 59.9% soymilk + 0.1% *Moringa* root powder; E = 99.9% soymilk + 0.1% *Moringa R*oot powder.

The final products were obtained as follows:

Into filtered whole cow milk, freshly prepared soymilk as per the formulations (Table 1) and a constant amount (0.1% of *Moringa* root powder) was added to each of the samples B, C, D, and E excluding sample A which served as the control.

The mixture was placed in a glass container and mixed with a glass rod. The blend (cow milk‐soymilk‐*Moringa*) was then pasteurized at 85 °C for 5 min and then rapidly cooled to 48 °C.

Inoculation with 3% w/v of yoghurt containing *Streptococcus thermophilus* and *Lactobacillus delbrueckii* ssp. *bulgaricus* was done. The inoculated mix was homogenized and incubated at 40–45 °C for 3–4 h for each and every lot. Timing was done with the help of a stop watch.

Each lot was then stirred and poured into PET bottles and stored at refrigeration temperature (6 °C) for 8 d.^[^ ^15^ ^]^

During storage the lots were checked for off odor (onset of spoilage) that was noticed at two weeks of refrigeration.

#### 2.2.4 Sensory Evaluation

The products were subjected to a sensory test using 45 panelists. Each attribute was scored based on its intensity scaled on a nine‐point hedonic scale (1 = disliked extremely; 2 = disliked very much; 3 = disliked moderately; 4 = disliked slightly; 5 = neither liked or disliked; 6 = liked slightly; 7 = like moderately; 8 = liked very much; 9 = liked very extremely) for color, texture, taste, flavor, and overall acceptability.

### 2.3 Determination of pH and Titratable Acidity

#### 2.3.1 pH

The pH of the soymilk and soy‐yoghurt samples was measured directly using an appropriate pH meter. The pH meter must be standardized with pH 4.0, 7.0, and 9.0 buffer solution before use.

#### 2.3.2 Titratable Acidity

The lactic acid content of soymilk and soy‐yoghurt samples was determined according to the Association of Official Analytical Chemists (AOAC)^[^ ^16^ ^]^ technique. Twenty grams of well homogenized sample is placed in beaker and was titrated against 0.1 n NaOH with phenolphthalein as indicator. Titratable acidity is expressed as percent lactic acid.

#### 2.3.3 Proximate Composition

The moisture content, ash, fat, protein, and all the samples were determined according to the method of ref. ^[^ ^17^ ^]^. All samples were analyzed in triplicate. Total carbohydrate content was calculated by difference.

Energy value (kcal/100 g) = 4 kcal × protein content + 4 kcal × carbohydrate content + 9 kcal × fat content.

### 2.4 Mineral Determination

The mineral content (calcium, magnesium, sodium, potassium, iron copper, zinc, and manganese) was determined according to the standard methods of the AOAC,^[^ ^18^ ^]^ using an atomic absorption spectrometer (Varian 220FS Spectr AA, Les Ulis, France). The sample was ashed at 550 °C and the ash boiled with 10 mL of 20% HCl in a beaker and then filtered into a 100 mL standard flask. All samples were analyzed in triplicate.

### 2.5 Statistical Analyses

Results were given as mean ± standard deviation and difference between means analyzed using the analysis of variance while differences within means were analyzed using Turkey test performed using statistical package for social science for Windows version 20.0 software. All these tests were carried out at *p* < 0.05.

## 3 Results and Discussion

### 3.1 Sensory Evaluation

The sensory evaluation of the different samples is presented in **Table** **2**.

**Table 2:** Sensory evaluation of the different samples

Samples	Color	Taste	Flavor	Texture	Overall acceptability
A (Control)	7.66 ± 0.88^a)^	7.4 ± 1.03^a)^	7 ± 1.03^a)^	6.80 ± 1.15^a)^	7.46 ± 0.62^a)^
B	6.6 ± 1.10^b)^	6.23 ± 1.0^b)^	6 ± 0.98^b)^	6.16 ± 0.91^b)^	6.36 ± 1.29^b)^
C	6.4 ± 0.93^b)^	6.56 ± 0.97^b)^	6.23 ± 0.93^b)^	5.90 ± 0.95	6.16 ± 0.83^c)^
D	5.96 ± 0.80^c)^	5.76 ± 0.81^c)^	5.4 ± 0.62^c)^	5.60 ± 0.93^c)^	5.6 ± 0.77^c)^
E	5.2 ± 0.55^c)^	5.13 ± 0.43^c)^	5.3 ± 0.59^c)^	5.13 ± 0.43^c)^	5.1 ± 0.40^c)^

^a–c)^ Mean values in the same column with different superscript letters are significantly different (*P* < 0.05). A = 100% cow whole milk + 7.5 g sugar/100 mL ( control); B = 60% whole cow milk + 39.9% soy milk + 0.1% *Moringa* root powder; C = 50% whole cow milk + 49.9% soymilk + 0.1% *Moringa* root powder; D = 40% whole cow milk + 59.9% soymilk + 0.1% *Moringa* root powder; E = 99.9% soymilk + 0.1% *Moringa R*oot powder.

Substitution significantly decreased color of samples (*p* < 0.05). B was similar to C and D was also similar to E. Color ranged from 5.2 (E) to 7.66 (A). The values were lower compared to values (8.65–9) found by Jayalalitha et al.^[^ ^19^ ^]^ Color is an important characteristic in the choice of yoghurt products. Sample A (control) was the most appreciated followed by the soymilk moringa enriched yoghurts (B and C) with increasing soy milk substitution. Samples D and E were the least preferred.

Substitution also significantly decreased taste of samples (*p* < 0.05). B was similar to C and D was also similar to E. Taste had the lowest score 5.13 (E) and highest score of 7.4 (A). The values were lower compared to values (8.65–9) found by Jayalalitha et al.^[^ ^19^ ^]^ Taste can affect consumer's choice for yoghurt products as B and C were the soy and Moringa enriched samples preferred to D and E with higher soymilk substitution.

Substitution significantly decreased also flavor of samples (*p* < 0.05). B was similar to C and D was also similar to E. Flavor ranged from the lowest score 5.3 (E) to highest score 7 (A).

Substitution also significantly decreased also texture of samples (*p* < 0.05). B was similar to C and D was also similar to E. Texture ranged from 5.13 (E) to 6.80 (A). The values were higher compared to (4.21–5) found by Javad et al.^[^ ^20^ ^]^ Texture is a key attribute that distinguishes yoghurt from other related products and is vital for consumers’ preference.

Substitution also significantly decreased overall acceptability of samples (*p* < 0.05). B was similar to C and D was also similar to E. The overall acceptability ranged from 5.1 (E) to 7.46 (A). According to above results, two other soy‐milk *Moringa* enriched yoghurts (B and C) were preferred after choosing the control (A) as the best. This indicates that soy‐milk yoghurt can be flavored with *Moringa* and become a good commercial product.

### 3.2 pH and Titratable Acidity of Soy‐Milk Yoghurt

**Table** **3** shows the pH and titratable acidity of the most appreciated samples.

**Table 3:** pH and titratable acidity of the most appreciated samples

Samples	pH	Titratable acidity (% lactic acid)
A	4.36 ± 0.02^a)^	0.52 ± 0.04^c)^
B	3.66 ± 0.01^b)^	0.35 ± 0.04^ab)^
C	4.11 ± 0.01^c)^	0.41 ± 0.04^a)^

^a–c)^ Mean values in the same column with different superscript letters are significantly different (*P* < 0.05). A = 100% cow whole milk + 7.5 g sugar/100 mL ( control); B = 60% whole cow milk + 39.9% soy milk + 0.1% *Moringa* root powder; C = 50% whole cow milk + 49.9% soymilk + 0.1% *Moringa* root powder.

The results obtain from Table 3 shows that the pH of the samples analyzed was significantly different (*p* < 0.05) from each other with sample A having the highest pH value of 4.36 and sample B having the lowest pH value of 3.66. These values were compared to those (3.6–4.3) found by Ponka et al.^[^ ^21^ ^]^ in traditional yoghourt consumed in Maroua‐Camaroon.

The titratable acidity ranged from 0.35 (C) to 0.52 (% lactic acid) (A). There was a significant difference (*p* < 0.05) between the three samples. Ukwo and Edima‐Nyah^[^ ^22^ ^]^ reported that titratability of yoghurt samples decreases as the level of soymilk substitution increases.

### 3.3 Proximate Composition of the Different Most Appreciated Samples

**Table** **4** shows the proximate composition of the most appreciated samples.

**Table 4:** Proximate composition of the different most appreciated samples

	A	B	C
Moisture [g/100 g FW]	70.86 ± 0.46^b)^	66.47 ± 1.26^a)^	72.46 ± 0.07^c)^
Ash [g/100 g DM]	1.32 ± 0.16^a)^	1.92 ± 0.15^c)^	1.62 ± 0.1^b)^
Fats [g/100 g DM]	3.29 ± 0.07^a)^	11.64 ± 0.16^b)^	12.35 ± 0.22^c)^
Crude protein [g/100 g DM]	30 ± 0.75^a)^	31.82 ± 1.42^b)^	32.47 ± 2.18^c)^
Crude fiber [g/100 g DM]	0 ± 0^a)^	2.09 ± 0.13^b)^	2.20 ± 0.12^b)^
Carbohydrates [g/100 g DM]	65.38 ± 0.83^c)^	50.51 ± 1.54^a)^	54.85 ± 2.13^b)^
Energy [kcal/100 g DM]	411.19 ± 1.01^a)^	442.16 ± 1.96^b)^	448.47 ± 2.22^c)^

^a–c)^ Mean values in the same line with different superscript letters are significantly different (*P* < 0.05). A = 100% cow whole milk + 7.5 g sugar/100 mL ( control); B = 60% whole cow milk + 39.9% soy milk + 0.1% *Moringa* root powder; C = 50% whole cow milk + 49.9% soymilk + 0.1% *Moringa* root powder.

The moisture content in the preferred samples ranged from 66.77 (B) to 72.46/100 g fresh weight (FW) (C) and was significantly different (*P* < 0.05).The moisture content depends on the amount of water evaporated during processing such as during pasteurization. The moisture content was also lower compared to the range (80–86/100 g FW) obtained by Akuso and Wordu,^[^ ^12^ ^]^ for most yoghurts.

The ash content ranged from 1.32 (A) to B (1925/100 g dry matter (DM)) and was significantly different (*P* < 0.05). The values were higher compared to 0.91/100 g DM found by Akuso and Wordu^[^ ^12^ ^]^ ( in most yoghurt. The ash content reflects the amount of minerals (macro‐ and micominerals), thus an increase in ash content purportedly analyzed to be caused by the addition of *Moringa* root powder suggests the possibility of using *Moringa* root powder as a food supplement in increasing the micronutrients of food hence an agent for food enrichment.

Substitution significantly increases the fat content in the samples (*P *< 0.05). The fat content varied from 3.29 (A) to 12, 35/100 g DM (C). This increase can be due to the presence of soy. The highest value seen in sample C (12.35/100 g DM) was compared to 12.40 g/100 g DM found by Kibui et al.^[^ ^23^ ^]^ in a related study.

The crude protein content increased from 30/100 g DM (A) to 33.82/100 g DM (B) and was significantly different (*p* < 0.05). The substitution with soymilk that is rich in protein and enrichment with *Moringa* root powder increased the protein content. Protein is important in providing firmness to yoghurt as it is seen in the process of standardization where protein content is increased to obtain a better texture and smoothness of the final yoghurt. However A had a better texture may be as a result of the presence of fiber limiting caseination.

Substitution significantly increases the fiber content in the samples (*P *< 0.05). There was no fiber present in A since it was 100% cow milk and there is no fiber from animal source. Due to the addition of soy milk and *Moringa* root powder in B and C. The crude fiber content was higher compared to the value (0.6/100 g DM) obtained by Kibui et al.^[^ ^23^ ^]^ in a similar study. Dietary fiber is important in increasing the functional properties of yoghurt. Fiber helps regulate peristalsis and is important for gut health. The recommended daily fiber for children and adults is 14 g/100 kcal and enriching yoghurt with soy milk and *Moringa* root powder help in contributing some of it.

The carbohydrate content decreased from 65.385/100 g DM (A) to 50.515/100 g DM (B) and the difference was significant (*p* < 0.05). However, the carbohydrate content increased significantly (*p* < 0.05) from 50515/100 g DM (B) to 54.85/100 g DM (C). The absence of lactose in soymilk and *Moringa* root powder which is rather present in milk. Thus the substitution of cow milk with soymilk could have reduced the amount of carbohydrates by reducing the amount of lactose.

The energy value ranged from 411.18 Kcal/100 g DM (A) to 448.47.5 Kcal/100 g DM (C). The energy value was higher compared to the value (157.87 Kcal/100 g DM) obtained by Kibui et al.^[^ ^23^ ^]^

### 3.4 Mineral Compositions of the Different Most Appreciated Samples

**Table** **5** shows the mineral compositions of the most appreciated samples. The amount of copper in the preferred yoghurt formulations ranged from 0.19 mg/100 g DM (A) to 0.31 mg/100 g DM (C) and was significantly different (*P* < 0.05).The increase in copper could be the result of supplementation with soymilk and *Moringa* root powder. Such increase in copper was also seen in a similar study involving the vegetable supplementation of regular yoghurt where values ranged from 0.36 mg/100 g DM (control yoghurt with no supplementation) to 0.43 mg/100 g DM with increasing percentage vegetable supplementation.^[^ ^24^ ^]^ Copper is an important component of enzymes involved in iron metabolism. Copper deficiency can cause normocytic, hypochromic anemia, leucopenia, and neuropenia and inclusive osteoporosis in children. There are however adverse effect of excessive consumption (>100 mg d^−1^) such as gastrointestinal distress and liver damage.^[^ ^25^ ^]^

**Table 5:** Min*e*ral composition (mg/100 g DM) the different most appreciated samples

Mineral content	A	B	C
Cu	0.19 ± 0.01^a)^	0.29 ± 0.01^b)^	0.31 ± 0.01^c)^
Mn	0.24 ± 0.01^a)^	0.35 ± 0.01^b)^	0.35 ± 0.01^b)^
Fe	6.36 ± 0.01^a)^	8.76 ± 0.01^b)^	13.53 ± 0.01^c)^
Zn	4.71 ± 0.01^c)^	3.25 ± 0.01^b)^	3.08 ± 0.01^a)^
Ca	649 ± 1^c)^	501 ± 1^b)^	382 ± 1^a)^
Mg	73 ± 0.5^c)^	61 ± 0.5^b)^	49 ± 0.5^a)^
K	607 ± 1^c)^	484 ± 1^b)^	399 ± 1^a)^
Na	491 ± 1^c)^	379 ± 1^b)^	271 ± 1^a)^

^a–c)^ Mean values in the same line with different superscript letters are significantly different (*P *< 0.05). A = 100% cow whole milk + 7.5 g sugar/100 mL ( control); B = 60% whole cow milk + 39.9% soy milk + 0.1% *Moringa* root powder; C = 50% whole cow milk + 49.9% soymilk + 0.1% *Moringa* root powder.

The manganese content ranged from 0.24 mg/100 g DM (A) to 0.35 mg/100 g DM (C) and the difference was significant (*P* < 0.05). Similar increase was registered in a related work where values ranged from 0.003 mg/100 g (control yoghurt with no supplementation) to 1.12 mg/100 g DM in the last treatment, upon increasing percentage vegetable supplementation and were similar. This suggests the possible importation of manganese from soymilk substitution and *Moringa* root powder supplementation. Values were higher than 0003 mg/100 g DM obtained by El‐karmany et al.^[^ ^24^ ^]^ and lower than 2.664 mg/100 g DM obtained by Kibui et al.^[^ ^23^ ^]^ The manganese content is desirable as increases bone health among humans as well as forms part of enzymes involved in amino acid, cholesterol, and carbohydrate metabolism.^[^ ^25^ ^]^ A deficiency in manganese will cause poor reproductive performance, growth retardation, abnormal function of bone and cartilage while excessive consumption will lead elevated blood concentration and neurotoxicity.^[^ ^25^ ^]^

The iron content of the yoghurt samples enriched with *Moringa* root powder significantly (*P* < 0.05) increased with increase of soymilk content in the formulation from 6.36 DM (A) to 13.53 mg/100 g DM (C). Soymilk is richer in iron than cow milk suggesting the soymilk substitution imported more iron as well as *Moringa* root powder addition. The values were higher compared to the value of 3.754 mg/100 g DM reported by Lovic et al.^[^ ^26^ ^]^ Desirable amounts of iron are important in the production of red blood cells as it forms the haem group of hemoglobin and an important constituent of enzymes. It thus helps to prevent microcytic hypochromic anemia and a deficiency will lead to pernicious anemia, impaired cognitive development, and impaired learning ability while excessive consumption will cause gastrointestinal distress.^[^ ^20^ ^]^

Zinc content decreased from 4.71 mg/100 g DM (A) to 3.08 mg/100 g DM mg/100 g DM (C) and the difference was significant (*P* < 0.05). Soymilk contains little or no zinc due to dehulling. Thus the increasing soymilk substitution led to decreasing amounts of zinc in the samples. These values were higher compared to 0.83 mg/100 g DM obtained by Kibui et al.^[^ ^23^ ^]^ Zinc is an important component of multiple enzymes and proteins, involved in the regulation of gene expression. Its deficiency will lead to growth retardation especially in children, loss of appetite, impaired immune function, hair loss, diarrhea, impotence, hypogonadism, and mental lethargy. However excessive consumption will lead to reduced levels of copper in the body, electrolyte imbalance, nausea and lethargy.^[^ ^25^ ^]^

Substitution significantly decreases the calcium content in the samples (*P *< 0.05). The calcium content ranged from 649 DM (A) to 382 mg/100 g DM (C). Those values were higher compared to 120 mg/100 g found by El‐karmany et al.^[^ ^24^ ^]^ The low calcium level may be explained by the lower amounts of calcium in soymilk compared to cow milk especially after dulling. Also the type of feeding could be a reason for decreased values of calcium in the cow milk. Calcium plays key roles in bone formation and mineralization. The calcium requirements during growth, pregnancy, and lactation are increased.^[^ ^27^ ^]^ Calcium plays an essential role in blood clotting, muscle contraction, nerve transmission, and bone and tooth formation. The lack of sufficient amounts of calcium will cause osteoporosis, hypertension, colon cancer, premenstrual syndrome, insulin resistance syndrome, and obesity while excess of it will cause the formation of kidney stones, hypercalcemia, milk alkali syndrome, and renal insufficiency.^[^ ^25^ ^]^

Substitution also significantly decreases the magnesium content in the samples (*P *< 0.05). Magnesium content varied from 73 mg/100 g DM (A) to 49 mg/100 g DM (C). However, the amounts were higher compared to the range of 32.5–39. 0 mg/100 g DM reported by Lovic et al.^[^ ^26^ ^]^ Magnesium is an important mineral in human nutrition. It is an important cofactor for enzyme systems hence its deficiency will lead to poor metabolism and consequently numbness, tingling, cramps, seizures, personality changes, abnormal heart rhythms, and coronary spams. Nonetheless it is not advisable to take in excessive amounts as it could lead to osmotic diarrhea and abdominal cramping.^[^ ^25^ ^]^ Substitution also significantly decreases the potassium content in the samples (*P* < 0.05).

Potassium content varied from 607 mg/100 g DM (A) to 399 mg/100 g DM (C). These amounts in the samples were higher than that obtained in a previous research 54.1 mg/100 g DM.^[^ ^28^ ^]^ Potassium helps the nerves to function and muscles to contract as well as helping with normal heartbeat, increasing iron utilization,^[^ ^29^ ^]^ and is beneficial to people taking diuretics to control hypertension who suffer from excessive excretion of potassium through the body fluid. Excessive intakes however will lead to hyperkalemia and possibly sudden death if excess is consumed by individuals with chronic renal insufficiency (kidney disease) or diabetes.^[^ ^25^ ^]^

Substitution also significantly decreases the sodium content in the samples (*P *< 0.05). Sodium content varied from 491 mg/100 g DM (A) to 271 mg/100 g DM (C). These values were lower than that obtained in a similar study by El‐karmany et al.^[^ ^24^ ^]^ Sodium helps maintain fluid volume outside of cells and thus normal cell function. It is beneficial for those suffering from general weakness and apathy, nausea and cramps in the muscles of the extremities, confusion, loss of reflexive movement, convulsions, or even coma. Excessive intake increases the risk of hypertension, cardiovascular diseases.^[^ ^25^ ^]^

## 4 Conclusion

Soy‐milk *Moringa* enriched yoghurts (B and C) were preferred after choosing the control (A) as the best. The incorporation of the soy‐milk *Moringa* significantly increases the fat, fiber, protein, copper manganese, and iron content in the samples. Thus moringa enriched soy‐milk yoghurts (B and C) will act as a cheaper alternative to the control (A) providing protein‐energy for low income families that will help reduce the occurrence of kwashiorkor and wasting. The presence of zinc and calcium will help in bone growth and development hence preventing stunting in children under 5 years and iron will help reduce the prevalence of anemia among pregnant women.

## Conflict of Interest

The authors declare no conflict of interest.

## Data Availability

The data that support the findings of this study are available from the corresponding author upon reasonable request.

## References

[1] The Future of Food and Agriculture, The Food and Agriculture Organization, 2017.

[2] Annual Evaluation Report, World Food Program, 2016.

[3] F. F. A. Anyangwe , CTA Working Paper , 2017, p. 17/08.

[4] WHO , http://www.who.org, (accessed: July 2021).

[5] UNICEF , A Framework for Child Sensitive Social Protection, 2019, p. 96.

[6] O. Rachmaniah , S. Kusumahati , E. O. Ningrum , F. Kurniawansyah , S. Zulaikah , H. Ni'Mah , S. Soeprapto , S. Soeprijanto , Conf. Ser. Mater. Sci. Eng. 2019, 543, 01.

[7] E. M. Outi , W. Viivi , E. Zannini , K. A. Elke , Crit. Rev. Food Sci. Nutr. 2015.

[8] S. L. Huffman , E. G. Piwoz , S. A. Vosti , K. G. Dewey , Matern. Child Nutr. 2014, 10, 562. 24847768 10.1111/mcn.12126 PMC4299489

[9] S. V. Karuna , N. Rajni , J. Nat. Prod. Plant. Resour. 2014, 4, 1.

[10] W. Braide , C. R. Ibegbulem , S. A. Adeleye , E. E. Anosike , P. B. Lugbe , L. J. Akien‐All , B. C. Uzor , M. C. Korie , Int. Jr. Res. Pharm. Biosci. 2017, 12, 4.

[11] Z. Nozipho , Master Thesis , University of Kwazulu‐Natal, South Africa 2016.

[12] O. M. Akusu , G. O. Wordu , Indian J. Nutr. 2017, 04, 04.

[13] RGPH , Recensement Général des Populations et de l'Habitat, 3ème Rapport d'OMD par le bureau central de recensement de la population (BUCREP) 2010.

[14] **CTA** , CTA Pro‐Agro collection , 2016.

[15] O. F. Osundahunsi , D. Amosu , B. O. T. Ifesan , Am. J. Food Technol. 2007, 2, 4.

[16] AOAC , Official Methods of Analysis, 16th ed., Association of Official Analytical Chemists, Washington DC 1990.

[17] AOAC , Official Methods of Analysis International, 17th ed., Association of Official Analytical Chemists, Washington DC 2000.

[18] AOAC , Official Methods of Analysis of the Association of Analytical Chemists International, 18th ed., Association of Official Analytical Chemists, Gathersburg, MD, USA 2005.

[19] V. Jayalalitha , A. P. Manoharan , B. Balasundaram , A. Elango , J. Nutr. Food Sci. 2015, 5, 427.

[20] G. A. Javad , S. T. Masoud , A. Amir , Int. J. Farming Allied Sci. 2016, 5, 6.

[21] R. Ponka , E. Beaucher , E. Fokou , G. Kansci , M. Piot , J. Leonil , F. Gaucheron , Afr. J. Biotechnol. 2013, 12, 49.

[22] S. P. Ukwo , Niger. J. Agric. Environ. 2015, 19, 11.

[23] A. N. Kibui , E. Owaga , M. Mburu , Afr. Jr. Food Agric. Nutr. Dev. 2018, 18, 1.

[24] A. M. El‐Karmany , M. F. Abel‐Ela , A. M. Hassanein , Jr. Food dairy Sci. 2013, 4, 4.

[25] M. Santoso , E. Damastuti , W. D. Ariyani , S. Kurniawati , W. Y. Yahfitri , At. Indones. 2011, 37, 2.

[26] M. Løvic , R. Marchelli , A. Martin , B. Moseley , Eur. Food Saf. Auth. 2009, 3, 996.

[27] A. Oscar , N. Stmin , M. R. Robert , Am. J. Clin. Nutr. 2004, 1, 80.

[28] H. Amellal‐chibane , S. Benamara , Am. J. Food Nutr 2011, 1, 1.

[29] C. M. Elinge , A. Muhammad , F. A. Atiku , A. U. Itodo , I. J. Peni , O. M. Sanni , Int. J. Plant Res. 2012, 13, 2.

